# Electrifying the case review process for better speed, reach, and impact

**DOI:** 10.1017/ash.2023.363

**Published:** 2023-09-29

**Authors:** Jennifer Gutowski, Melissa Bronstein, Adam Tatro, Stephany Frey, Emil Lesho

## Abstract

**Background:** Prevention of healthcare-associated infections (HAIs) requires timely feedback to and input from all staff involved in patient care to best identify practice gaps and improvement targets. However, multidisciplinary review of HAI events can be challenging to promptly complete given staffing shortages and the excess administrative burden of emailed and printed forms and disjointed analyses, reporting, and visualization tools. Plagued by a lack of feedback from attending and ordering physicians, difficulty transcribing and analyzing nonstandardized data, and challenges in summarizing and distributing actionable findings, we sought to reduce turnaround time (TAT), improve data collection, and broaden communication of HAI contributing factors and proposed solutions. **Methods:** A secure web application for electronic data capture and reporting, Research Electronic Data Capture (REDCap), was used; the software application is free to nonprofit organizations. The review process is now initiated by an infection preventionist entering HAI information into an initial survey, which automatically cascades information into 4 subsequent surveys, distributed through automated email links, providing an opportunity for individual responses from the nursing unit, the attending provider, an infectious disease physician, and the ordering provider for the positive test that detected the HAI. Survey questions focus on evaluation of adherence to CDC and SHEA HAI prevention strategies. Reminders are automatically generated and continue to be sent to involved staff until their portion is completed. Survey responses are automatically summarized upon completion of all reviews and are shared with several stakeholders, including hospital leadership, the care team, infection prevention staff, and quality-control partners (Fig.). Discrete qualitative and quantitative data are exported in a standard application-programming interface (API) format for immediate analysis and interpretation. **Results:** After the review process was launched using new electronic technology, the average TAT and completion rate improved from 23 days and 40% to 7 days and 95%, respectively. Input from ordering and attending physicians, once extremely rare, became frequent. Nuanced insight into causative and preventive factors, previously unachievable, occurred during review of all 38 HAIs reported in December 2022. Reviewers believed that 48% of HAIs reviewed could have been prevented. **Conclusions:** Applying electronic technology to HAI case review improved completion and timeliness of reviews by both providers and nurses. By sharing data and insights with all stakeholders in real time, the new procedure permitted multidirectional communication between the care teams and increased awareness of patient harm as well as ownership of patient safety. Our process is freely and readily generalizable to any nonprofit healthcare facility.

**Figure:**
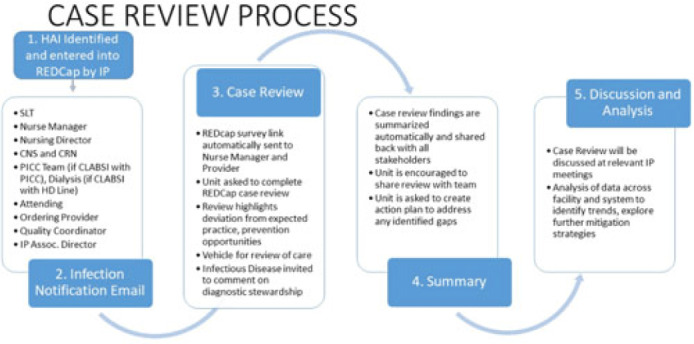

**Disclosures:** None

